# Assessing the therapeutic potential of vagus nerve stimulation in autoimmune diseases: A systematic review

**DOI:** 10.14814/phy2.70230

**Published:** 2025-02-04

**Authors:** Eubi Chan, Ali R. Mani

**Affiliations:** ^1^ Network Physiology Laboratory UCL Division of Medicine London UK; ^2^ School of Medicine Brighton and Sussex Medical School Brighton UK

**Keywords:** autoimmune disease, immunomodulation, vagus nerve stimulation

## Abstract

Emerging evidence suggests that the vagus nerve can modulate the immune system in experimental settings. Vagus nerve stimulation (VNS), initially developed for managing epilepsy, is now being explored as a treatment for autoimmune diseases due to its potential immunomodulatory effects. This systematic review evaluates the therapeutic potential of VNS in autoimmune diseases by critically appraising findings from human clinical studies. This systematic review was conducted in accordance with the PRISMA guideline, with a comprehensive literature search performed in Ovid, Cochrane, and PubMed databases up to July 2024. Studies focusing on VNS in patients with autoimmune diseases were eligible, and the quality of study was assessed using the QualSyst tool. Of the 53 papers identified for full‐text assessment, 19 studies met the eligibility criteria. Findings suggest that VNS is a promising adjunctive therapy for Crohn's disease and rheumatoid arthritis, showing potential to alleviate symptoms and modulate immune responses. The efficacy and safety of VNS vary widely across studies, highlighting the complex nature of autoimmune diseases and the diverse mechanisms of VNS action. Future research should prioritize large‐scale, randomized controlled trials with standardized protocols to further elucidate the efficacy, long‐term safety, and optimal parameters of VNS across various autoimmune conditions.

## 1 INTRODUCTION

Autoimmune diseases, characterized by the immune system's aberrant attack on the body's own tissues, pose a significant clinical challenge due to their chronic nature and complex pathophysiology. Rheumatoid arthritis (RA), Crohn's disease (CD), multiple sclerosis (MS), and type 1 diabetes mellitus (T1DM) are among the autoimmune diseases affecting millions worldwide, contributing to high morbidity and diminished quality of life (Campbell, 2014; Conrad et al., 2023). Current therapeutic approaches primarily focus on alleviating symptoms and modulating immune responses. However, many patients continue to experience inadequate disease control and treatment‐related side effects, underscoring the need for novel therapeutic strategies (Fugger et al., 2020; Shams et al., 2021).

Vagus nerve stimulation (VNS) has emerged as a promising therapeutic approach for autoimmune diseases. Initially developed for epilepsy and later approved for treatment‐resistant depression (Nemeroff et al., 2006; Uthman et al., 2004), VNS has garnered attention for its role in modulating the immune system through the cholinergic anti‐inflammatory pathway (Pavlov & Tracey, 2017; Tracey, 2009). This pathway leverages the vagus nerve's capacity to reduce the production of pro‐inflammatory cytokines through activation of α_7_‐nicotinic cholinergic receptors, thereby attenuating inflammatory responses without compromising overall immune function (Pavlov & Tracey, 2017; Tracey, 2009). However, the exact mechanism by which VNS modulates the immune system is not well understood and may involve the activation of brain regions responsible for immune regulation (Jin et al., 2024) via stimulation of vagal afferent fibers.

VNS can be delivered via two primary methods: invasive VNS (iVNS) and transcutaneous VNS (tVNS). iVNS involves the surgical implantation of a pulse generator connected to the left cervical vagus nerve, while tVNS provides a noninvasive alternative by delivering electrical stimulation through the skin, targeting either the auricular branch in the outer ear or the cervical region of the neck (Howland, 2014; Shao et al., 2023). While iVNS has demonstrated efficacy, it carries surgical risks such as infection and device‐related complications. In contrast, tVNS offers a more convenient and safer option, although its therapeutic protocols are less standardized, necessitating further research to optimize its application across various clinical indications.

Preclinical studies and clinical trials have supported the potential of VNS in managing autoimmune diseases. For example, in animal models, VNS has demonstrated the ability to modulate peripheral immune responses and reduce disease severity in conditions such as inflammatory bowel diseases (IBD) and RA (Caravaca et al., 2022; Levine et al., 2014; Meregnani et al., 2011). Similarly, multiple human studies have reported improvements in patient‐reported outcomes in conditions such as CD (Bonaz et al., 2016; D'Haens et al., 2023; Sinniger et al., 2020) and RA (Drewes et al., 2021; Koopman et al., 2016; Marsal et al., 2021), further highlighting the potential of VNS to enhance the quality of life for patients with autoimmune conditions.

Despite these promising findings, the application of VNS in autoimmune diseases remains in its early stages, with many unanswered questions regarding its mechanisms of action, optimal stimulation parameters, and long‐term efficacy. The diversity of autoimmune diseases further complicates the translation of VNS as a universal treatment modality, necessitating a nuanced understanding of disease‐specific responses to vagal modulation.

This systematic review aims to critically assess the current evidence on the therapeutic potential of VNS in autoimmune diseases by synthesizing findings from all published human studies. The primary objective is to evaluate the efficacy and safety of VNS in autoimmune diseases, identifying gaps in knowledge to inform future research and clinical practice. Specifically, this review will:

1. Analyze intervention protocols, including stimulation parameters, duration, and frequency.
2. Examine the therapeutic efficacy of VNS on primary and secondary clinical outcomes across different autoimmune diseases.
3. Evaluate the safety profile of VNS in the context of autoimmune diseases.
4. Provide guidance for clinical practice and inform future research directions.

## 2 METHODS

This systematic review was conducted in accordance with the PRISMA guideline (Moher et al., 2009). This review explores the therapeutic effects of VNS as an intervention in autoimmune diseases, incorporating studies published up to July 19, 2024.

### 2.1 Eligibility criteria

Studies were eligible for inclusion if the following criteria were met: (1) participants with autoimmune diseases; (2) iVNS or tVNS therapy; (3) original research articles, including randomized controlled trials (RCTs), pilot studies, observational studies (prospective or retrospective), case reports, and case series. No restrictions were imposed based on the participants' age, gender, or the year of publication. Only studies published in English were included. Studies without detailed stimulation parameters were also included to provide a broader context of the clinical populations and interventions studied.

The following types of literature were excluded: reviews, conference abstracts, poster presentations, editorials, commentaries, protocols, and gray literature (e.g., dissertations). Additionally, experimental studies that applied VNS in animal models or healthy individuals were excluded, focusing the review solely on clinical applications in human autoimmune disease populations.

### 2.2 Information sources

A comprehensive search was performed across three major databases: Ovid, Cochrane Library, and PubMed, from their inception to July 19, 2024. Both forward and backward citation tracking, along with manual searches, were used to ensure a thorough identification of relevant studies. The search strategy combined free‐text terms and subject headings using the format [intervention] AND [disease] to capture a wide range of relevant studies (see Table 1). A filter for human studies was applied in Ovid to exclude animal research. The search included full‐text articles, with no restriction to titles or abstracts, ensuring a broad and inclusive approach to literature retrieval.

**TABLE 1:** Search terms used to identify papers related to vagus nerve stimulation and autoimmune diseases (Ovid).

Intervention	Disease
Vagus nerve stimulation	Autoimmune disease
Vagal nerve stimulation	Rheumatoid arthritis
VNS	Systemic lupus erythematosus
	Crohn's disease
	Ulcerative colitis
	Inflammatory bowel disease
	Systemic sclerosis
	Multiple sclerosis
	Type 1 diabetes

### 2.3 Study selection

**FIGURE 1:**
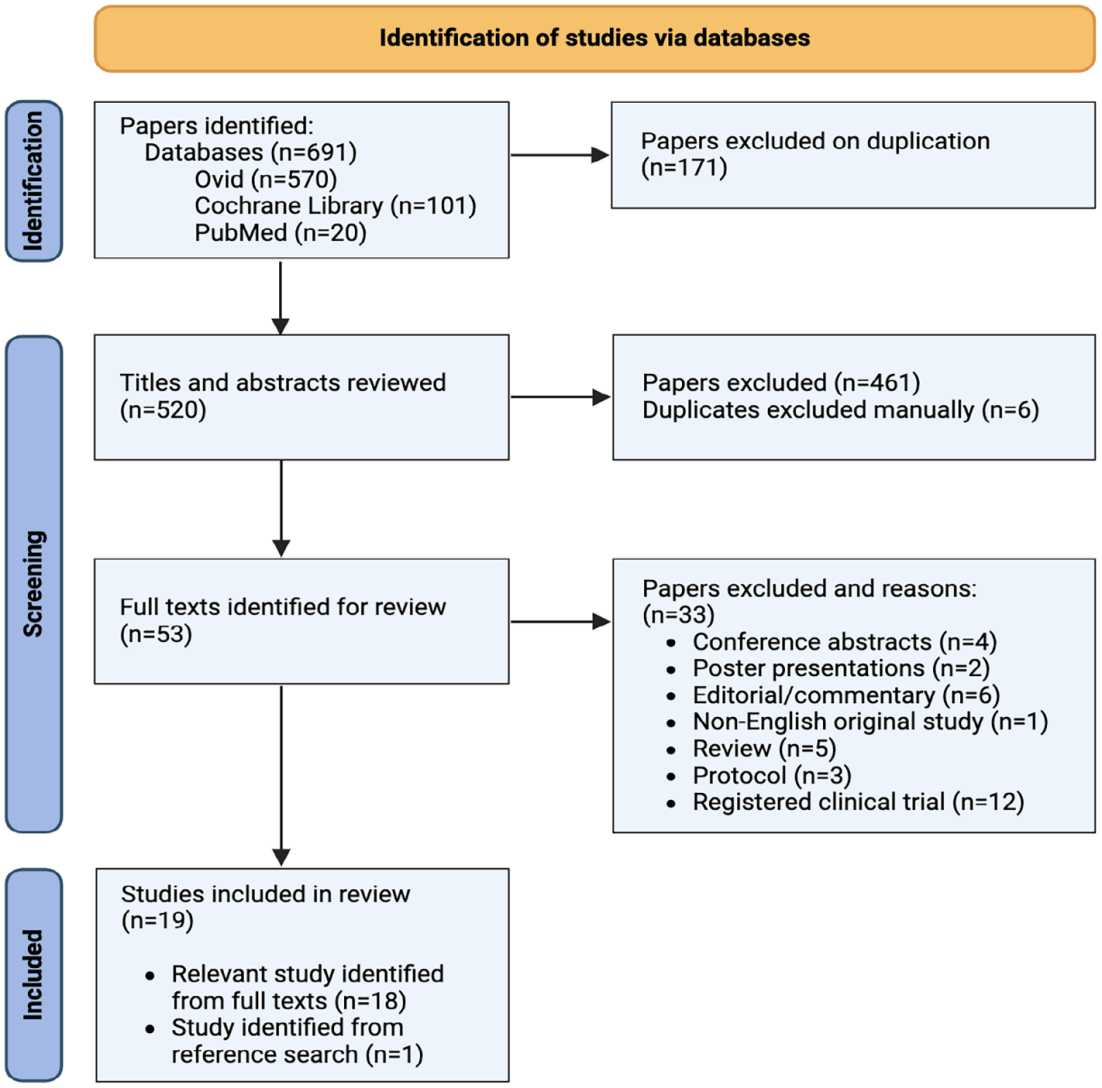
PRISMA flowchart of study selection.

The selection process involved an initial screening of titles and abstracts to identify studies that met the eligibility criteria. All reasons for exclusion were documented and illustrated in the PRISMA flow diagram (Figure 1). For studies with uncertain eligibility, discussions were held between two independent reviewers (Eubi Chan [EC] and Ali R. Mani [AM]) to reach a consensus. Full‐text reviews were subsequently conducted to confirm the inclusion of relevant studies, ensuring all decisions were made based on a comprehensive assessment of the available data.

### 2.4 Grading the quality of the studies

The quality of the included studies was independently assessed by two reviewers (EC and AM) using the Standard Quality Assessment Criteria for Evaluating Primary Research Papers (QualSyst) for quantitative studies. The QualSyst tool consists of 14 criteria, each scored on a scale from zero to two, with an option to mark an item as “not applicable” (NA). The total possible score is 28, with results expressed as a percentage of applicable items. Studies were categorized based on their quality scores: <50% as poor quality, 50%–69% as fair quality, 70%–79% as good quality, and >80% as strong quality (Kmet et al., 2020). Discrepancies between reviewers were resolved through discussion to ensure consistent evaluation of study quality. Appendix S1 provides a detailed summary of the quality assessment scores across the included studies.

### 2.5 Data extraction and synthesis

Data extraction was conducted using a standardized table to capture key study characteristics, including study design, participant demographics, intervention details (e.g., VNS parameters), primary and secondary outcome measures, and reported adverse events (AE). The extracted data were synthesized qualitatively due to the heterogeneity in study designs, intervention protocols, and outcome measures. Quantitative synthesis was considered inappropriate given the variability in the included studies. Therefore, a narrative synthesis was adopted to summarize the findings and identify key patterns. The therapeutic effect of VNS was assessed based on statistically significant improvements (*p* < 0.05) in primary clinical outcomes. In pre‐post studies, improvements were compared to pre‐treatment levels, while in RCTs, comparisons were made to control groups. Secondary outcomes were also considered, particularly where primary outcomes were nonclinical (e.g., safety of VNS), to provide a comprehensive evaluation of VNS's impact.

## 3 RESULTS

A total of 691 publications were identified through searches in three electronic databases (Ovid: *n* = 570; Cochrane Library: *n* = 101; PubMed: *n* = 20). After removing 171 duplicates, 520 papers remained and were screened against the eligibility criteria. Of these, 467 did not meet the criteria and were excluded, including six duplicates identified through manual review. The full texts of the remaining 53 articles were assessed, resulting in the exclusion of 33 papers for the following reasons: conference abstracts (*n* = 4), poster presentations (*n* = 2), editorials or commentaries (*n* = 6), non‐English studies (*n* = 1), reviews (*n* = 5), protocols (*n* = 3), and registered clinical trials with unpublished peer‐reviewed results (*n* = 12). One additional study was identified through manual reference searching. Ultimately, 19 studies met the eligibility criteria and were included in this review. These comprised 8 RCTs (Aranow et al., 2021; Baker et al., 2023; Bellocchi et al., 2023; Genovese et al., 2020; Kornum et al., 2024; Marrosu et al., 2007; Peterson et al., 2024; Sahn et al., 2023; Tarn et al., 2023), nine prospective studies (Addorisio et al., 2019; Bonaz et al., 2016; D'Haens et al., 2023; Drewes et al., 2021; Jensen et al., 2022; Kibleur et al., 2018; Koopman et al., 2016; Marsal et al., 2021; Sinniger et al., 2020), one case report (Clarençon et al., 2014), and one case series (Marrosu et al., 2007). Figure 1 provides a flowchart summarizing the study selection process.

### 3.1 Characteristics of included studies

Table 2 summarizes the characteristics of the 19 included studies published between 2007 and 2023, each of which investigated the therapeutic potential of VNS in autoimmune diseases. These studies were conducted across multiple countries, including France (21%), Denmark (21%), the USA (15.8%), Italy (10.5%), and others. The studies covered a wide range of autoimmune conditions, including CD, IBD, RA, MS, T1DM, systemic lupus erythematosus (SLE), systemic sclerosis (SSc), and Primary Sjögren's syndrome (PSS). Sample sizes ranged from 1 to 131 participants, with participant completion rates (i.e., the number of participants who completed all study‐related activities, including interventions and follow‐ups) varying from 1 to 118. Participant ages ranged from 10 to 85 years.

**TABLE 2:** Characteristics of selected studies.

Author (year)	Disease	Country	Study type	Mean age, years (range)	Comparator	Gender (M/F)	Participants (VNS/sham)	Completed, *N*
D'Haens et al. (2023)	CD	The Netherlands	PS	35.4 (21–62)	Pre	13/4	16	15
Sinniger et al. (2020)		France	PS	39 (20–52)	Pre	5/4	9	7
Kibleur et al. (2018)		France	PS	38	Pre	NR	9	9
Bonaz et al. (2016)		France	PS	34.3 (20–51)	Pre	4/3	7	5
Clarençon et al. (2014)		France	CR	49	Pre	Male	1	1
Sahn et al. (2023)	IBD	USA	RCT	Median: 15 (10–21)	Sham taVNS	12/10	22 (12/10)	21 (11/10)
Peterson et al. (2024)	RA	USA	RCT	57.9	Sham iVNS	9/51	60	59
Baker et al. (2023)		USA	RCT	54.4 (18–75)	Sham taVNS	20/93	113 (61/52)	101
Marsal et al. (2021)		Spain	PS	54.4 (18–80)	Pre	3/27	30	27
Drewes et al. (2021)		Denmark	PS	HDA: 54 LDA: 58	Pre	5/31	56	36
Genovese et al. (2020)		Denmark	RCT	50.9 (26–73)	Sham iVNS	3/11	Stage 1: 3 Stage 2: 11 (7/4)	14
Addorisio et al. (2019)		USA & The Netherlands	PS	(28–70)	Pre	2/7	9	9
Koopman et al. (2016)		The Netherlands, Bosnia, Herzegovina, Croatia	PS	51 (36–69)	Pre	4/14	18	17
Jensen et al. (2022)	RA/SLE	Denmark	PS	57 (18–85)	Pre	12/40	52 RA (*n* = 47), SLE (*n* = 5)	52
Aranow et al. (2021)	SLE	USA	RCT	45.7	Sham taVNS	6/12	18 (12/6)	18 (12/6)
Bellocchi et al. (2023)	SSc	Italy	RCT	58	Sham taVNS	14/18	32	21
Marrosu et al. (2007)	MS	Italy	CS	32	Pre	Male	3	3
Kornum et al. (2024)	T1DM (DGP)	Denmark	RCT	54 (20–86)	Sham taVNS	53/78	131 (63/68)	116 (59/57)
Tarn et al. (2023)	PSS	UK	RCT	59.6 (21–84)	Sham taVNS	4/36	40 (20/20)	30 (13/17)

Abbreviations: CD, Crohn's disease; CR, case report; CS, case series; DGP, diabetic gastroenteropathy; HDA, high disease activity; IBD, inflammatory bowel disease; LDA, low disease activity; M/F, male/female; MS, multiple sclerosis; N, number; NR, not reported; PS, prospective study; PSS, primary Sjögren's syndrome; pts., participants; RA, rheumatoid arthritis; RCT, randomized controlled trials; SLE, systemic lupus erythematosus; SSc, systemic sclerosis; T1DM, type 1 diabetes mellitus.

Regarding the comparator, 11 studies (57.9%) employed a pre‐post design, while eight studies (42.1%) were RCTs comparing VNS with a sham control group. Gender distribution varied by disease focus: studies on CD and IBD included a higher proportion of male participants, while studies on RA, SLE, SSc, T1DM, and PS were predominantly female.

None of the included studies were of poor quality according to the QualSyst criteria, and no study was excluded based on its quality (see Appendix S1).

### 3.2 Stimulation side and site, intervention, and technical features

Nine studies used iVNS, consistently targeting the left cervical vagus nerve (Bonaz et al., 2016; Clarençon et al., 2014; D'Haens et al., 2023; Genovese et al., 2020; Kibleur et al., 2018; Koopman et al., 2016; Marrosu et al., 2007; Peterson et al., 2024; Sinniger et al., 2020). In contrast, tVNS studies targeted both auricular and cervical regions. Four studies focused on the left auricular branch of the vagus nerve (cymba conchae) (Aranow et al., 2021; Bellocchi et al., 2023; Jensen et al., 2022; Sahn et al., 2023), one on the right (Addorisio et al., 2019), and three on the cervical vagus nerve (Drewes et al., 2021; Kornum et al., 2024; Tarn et al., 2023).

VNS devices varied across studies. Invasive interventions primarily employed the Cyberonics Model 302 or the SetPoint Medical device. tVNS studies employed devices such as NEMOS, TENS 7000, and GammaCore, each with distinct technical specifications. Pulse width ranged from 100 μs to 500 μs, and frequencies varied from 10 Hz in iVNS studies to 20–30 Hz in tVNS, with one study using a notably higher frequency of 20 kHz (Marsal et al., 2021). Current intensities also varied significantly, reflecting the experimental nature of these interventions. Table 3 provides a detailed summary of VNS stimulation side, site, intervention details, and technical specifications across studies.

**TABLE 3:** Summary of VNS stimulation side and site, intervention, and technical features.

Author (year)	Intervention	Device model	Stimulation side and site	Pulse width	Frequency (Hz)	Intensity (mA)	Stimulation period	Study duration
D'Haens et al. (2023)	iVNS	Cyberonics PerenniaFLEX Lead Model 304 [now LivaNova]	Left cervical VN	250 μs	10	0.25–2	1–5 min, 1–4× daily	4 months
Sinniger et al. (2020)		Cyberonics Model 302	Left cervical VN	250–500 μs	10	NR	30s ON and 5 min OFF, continuously	12 months
Kibleur et al. (2018)		Cyberonics Model 302	Left cervical VN	500 μs	10	0.5–1.25	30s ON and 5 min OFF, continuously	12 months
Bonaz et al. (2016)		Cyberonics Model 302	Left cervical VN	500 μs	10	0.25–1.25	30s ON and 5 min OFF, continuously	6 months
Clarençon et al. (2014)		Cyberonics Model 302	Left cervical VN	500 μs	10	0.5–1	30s ON and 5 min OFF, continuously	12 months
Koopman et al. (2016)		Cyberonics	Left cervical VN	250 μs	10	0.25–2	60s, 1–4x daily	84 days
Genovese et al. (2020)		SetPoint Medical	Left cervical VN	250 μs	10	0.1–2.5	1 min 1× daily, or 1 min 4× daily, or no stimulation (*n* = 4)	3 months
Peterson et al. (2024)		NR	Left cervical VN	250 μs	10	Max 2.5	60s daily	3 months
Marrosu et al. (2007)		NR	NR	250 μs	10	0.25–1.25	62 s ON and 60s OFF	26 months
Sahn et al. (2023)	taVNS	TENS 7000, Roscoe Medical	Left cymba conchae	300 μs	20	NR	5 min, 1x daily	4 months
Aranow et al. (2021)		TENS 7000, Roscoe Medical	Left cymba conchae	300 μs	30	NR	5 min, 1x daily	4 days
Baker et al. (2023)		Nēsos (formerly Vorso)	NR	NR	20	1–2.6	15 min, 1× daily	3 months
Marsal et al. (2021)		Nēsos	NR	NR	20 kHz	NR	Up to 30 min daily	3 months
Addorisio et al. (2019)		Brookstone	Right cymba conchae	NR	NR	250–280	5 min, 2× daily	2 months
Jensen et al. (2022)		NEMOS	Left cymba conchae	250 μs	25	0.5	30 s ON and 30 s OFF for 30 min, one session only	Single session
Bellocchi et al. (2023)		NR	Left cymba conchae	250 μs	25	0.2–5	30s ON and 30s OFF for 4 non‐consecutive hours	2 months
Kornum et al. (2024)	tcVNS	GammaCore	Bilateral cervical VN	NR	NR	1–40 AU	120 s 4× daily for 1 week, 120 s 2× daily for 8 weeks	22 months
Drewes et al. (2021)		GammaCore	Bilateral cervical VN	1 ms, bursts repeated once every 40 ms	25	Max 60	120 s, 3× daily	4 days
Tarn et al. (2023)		GammaCore	Bilaterial cervical VN	5 ms	25	Max 60	120 s per dose, 2× daily	54 days

Abbreviations: AU, arbituary units; iVNS, invasive vagus nerve stimulation; NR, not reported; taVNS, transcutaneous auricular vagus nerve stimulation; tcVNS, transcutaneous cervical vagus nerve stimulation; VN, vagus nerve.

Intervention durations ranged from a single session (Jensen et al., 2022) to 26 months (Marrosu et al., 2007), with most of the studies applying daily stimulation. This wide range in duration and cumulative VNS exposure could significantly influence observed outcomes, making direct comparisons between studies challenging.

### 3.3 Efficacy

The efficacy of VNS varied across studies. Significant improvements in primary outcomes, such as reductions in disease activity scores and pro‐inflammatory cytokines, were observed particularly in CD and RA. However, evidence for efficacy in other autoimmune conditions, including SLE, SSc, MS, and T1DM were less conclusive and showed mixed results. Table 4 provides a detailed summary of primary and secondary outcomes and their statistical significance across studies. In brief, three out of six studies examining the effect of VNS in CD used the Crohn's Disease Activity Index (CDAI) as the primary measure to assess the intervention's effects (Bonaz et al., 2016; D'Haens et al., 2023; Sinniger et al., 2020). CDAI is a clinical tool commonly used in clinical trials to evaluate the severity of symptoms and disease activity in CD patients. These three studies demonstrated improvements in disease severity. For example, D'Haens et al. recruited 16 patients with moderately to severely active CD and observed a significant decrease in CDAI. Additionally, they reported a significant reduction in fecal calprotectin (a protein found in neutrophils and a biomarker of intestinal inflammation), a decrease in mucosal inflammation in 11/15 patients with paired endoscopies, and a reduction in TNF‐α and interferon‐γ levels (D'Haens et al., 2023). In another study, after 1 year of VNS in nine patients with moderately active CD, five patients achieved clinical remission, and six patients reached endoscopic remission (Sinniger et al., 2020).

**TABLE 4:** Summary of primary and secondary outcomes and statistical significance.

Author (year)	Disease	Primary outcome measure (s)	Did VNS show a significant effect (*p* < 0.05) on primary outcome? Yes/No	Secondary outcome measure (s)	Did VNS show a significant effect (*p* < 0.05) on secondary outcome? Yes/No
D'Haens et al. (2023)	CD	CDAI	Yes	SES‐CD, FC, hsCRP, IBDQ SHS	Yes: FC
Sinniger et al. (2020)		CDAI	Clinical remission but no statistical analysis reported	CRP, FC, CDEIS, HRV, HAD, self‐reported pain (VAS), cytokine levels	No statistical analysis reported
Kibleur et al. (2018)		EEG changes	Yes	CRP, FC, HRV, HAD	Yes
Bonaz et al. (2016)		CDAI	Clinical remission but no statistical analysis reported	CRP, FC, CDEIS, HRV, HAD, self‐reported pain (VAS)	No statistical analysis reported
Clarençon et al. (2014)		EEG changes, HRV	Yes	CDAI	Yes
Sahn et al. (2023)	IBD	wpCDAI, PUCAI, FC	No p‐values provided for wpCDAI and PUCAI. Yes: FC in UC pts	PROMIS, HRV	No statistical analysis reported
Peterson et al. (2024)	RA	Safety (AE and SAE)	NA	Clinical efficacy and patient‐reported effectiveness out‐ comes will be presented in a forthcoming report.	NA
Baker et al. (2023)		ACR20 response	No	DAS28‐CRP, HAQ‐DI, ACR50 and ACR70 responses, CDAI, SDAI	Yes: HAQ‐DI
Marsal et al. (2021)		DAS28‐CRP	Yes	Safety (AE and SAE), ACR responses, HAQ‐DI	Yes: ACR responses and HAQ‐DI
Drewes et al. (2021)		DAS28‐CRP	Yes, in High DA group	CRP, CVT, cytokine levels	Yes: CRP and IFN‐γ in High DA group, CVT and IL‐10 in Low DA group
Genovese et al. (2020)		Safety (AE and SAE)	NA	DAS28‐CRP, CDAI, Synovitis	No
Addorisio et al. (2019)		TNF levels	Yes	IL‐6 and IL‐1β levels, DAS28‐CRP, VAS scores	Yes
Koopman et al. (2016)		DAS28‐CRP	Yes	TNF production, cytokine levels, ACR responses, EULAR response	Yes: TNF and IL‐6
Jensen et al. (2022)	RA or SLE	HRV parameters (SDNN, RMSSD, PNN50)	Yes	Comparison between deep breathing and taVNS	NA
Aranow et al. (2021)	SLE	Self‐reported pain VAS	Yes	Fatigue (FACIT‐F), tender and swollen joint counts, PtGA and PGA, inflammatory biomarkers and neuropeptides levels	Yes: FACIT‐F, tender and swollen joints, and plasma levels of substance P
Bellocchi et al. (2023)	SSc	NRS	Yes	Inflammatory biomarkers, HRQoL, HRV	Yes: IL‐6 levels
Marrosu et al. (2007)	MS	PCT	NR	Dysphagia improvement	NR
Kornum et al. (2024)	T1DM (DGP)	Gastrointestinal Symptoms (GCSI, GSRS)	No	Gastrointestinal transit times, CAN score, CVT	Yes: Gastric emptying time
Tarn et al. (2023)	PSS	Fatigue (PRO‐F and fVAS)	Yes	Neurocognitive function (measured using neurocognitive test), muscle bioenergetics, immune response, HRV	Yes: backward digit span test and IL‐6 levels

Abbreviations: ACR, American College of Rheumatology; AE, adverse events; CAN, cardiac autonomic neuropathy; CD, Crohn's disease; CDAI, Crohn's Disease Activity Index; CDEIS, Crohn's Disease Endoscopic Index of Severity; CRP, C‐reactive protein; CVT, cardiac vagal tone; DA, disease activity; DAS28‐CRP, disease activity score in 28 joints using C‐reactive protein; DGP, diabetic gastroenteropathy; EEG, electroencephalogram; EULAR, European League Against Rheumatism; FACIT‐F, functional assessment of chronic illness therapy fatigue; FC, fecal calprotectin; fVAS, visual analog scale of abnormal fatigue; GCSI, Gastroparesis Cardinal Symptom Index; GSRS, Gastrointestinal Symptom Rating Scale; HAD, hospital anxiety and depression; HAQ‐DI, Health Assessment Questionnaire‐Disability Index; HRQoL, health‐related quality of life; HRV, heart rate variability; hsCRP, high‐sensitivity C‐reactive protein; IBDQ, Inflammatory Bowel Disease Questionnaire; IL, interleukin; MS, multiple sclerosis; NA, not applicable; NR, not reported; NRS, Numeric Rating Scale; PCT, postural cerebellar tremor; PGA, physician global assessment; PRO‐F, profile of fatigue; PROMIS, patient‐reported outcomes measurement information system; PSS, primary Sjögren's syndrome; PtGA, patient global assessment; pts., participants; RA, rheumatoid arthritis; SAE, serious adverse event; SDAI, Simplified Disease Activity Index; SES‐CD, simple endoscopic score‐Crohn's disease; SHS, simple health score; SLE, systemic lupus erythematosus; SSc, systemic sclerosis; T1DM, type 1 diabetes mellitus; TNF, tumor necrosis factor; UC, ulcerative colitis; VAS, visual analog scale.

Three out of seven studies examining the effect of VNS on rheumatoid arthritis (RA) used the DAS28‐CRP score to assess disease activity and systemic inflammation (Drewes et al., 2021; Koopman et al., 2016; Marsal et al., 2021). These studies demonstrated improvements in disease severity following VNS. For example, in an uncontrolled open‐label study, Marsal et al. showed that VNS was well‐tolerated in RA patients (*n* = 30), with clinically meaningful reductions in DAS28‐CRP (Marsal et al., 2021). In another open‐label study, Drewes et al. investigated the effect of VNS in two cohorts of RA patients: one with high disease activity (*n* = 16) and one with low disease activity (*n* = 20). The results indicated that, in the high disease activity group, VNS led to reductions in DAS28‐CRP and serum interferon‐γ, while no significant effects were observed in the low disease activity group (Drewes et al., 2021). Koopman et al. employed a more complex study design to examine how switching VNS stimulation on and off affected RA patients (Koopman et al., 2016). In a study of 18 patients with RA, they observed a significant reduction in DAS28‐CRP from baseline to day 42 while the device was delivering electrical stimulation. However, when the device was turned off at day 42, DAS28‐CRP worsened significantly within 14 days. Restarting the device at day 56 resulted in a significant reduction in DAS28‐CRP (Koopman et al., 2016).

Only a few studies have investigated the effect of VNS in conditions such as SLE, SSc, MS, and T1DM, with mixed results (see Table 4).

### 3.4 Safety and adverse events

Adverse events (AE) associated with VNS were generally mild to moderate. The most commonly reported AE were voice alteration or hoarseness (21.1%), headache (21.1%), throat pain (10.5%), and skin irritation (10.5%). Serious adverse events (SAE) were rare, occurring in only two studies (10.5%), and primarily related to factors independent of VNS, such as pre‐existing autoimmune conditions (D'Haens et al., 2023; Peterson et al., 2024). Dropouts specifically attributed to VNS‐related side effects were reported in four studies. These included a postoperative wound infection following iVNS device implantation (D'Haens et al., 2023), transient headache with tcVNS (Drewes et al., 2021), hoarseness after active tcVNS (Kornum et al., 2024), and unspecified side effects from tcVNS (Tarn et al., 2023). Across the remaining 15 studies, no VNS‐related dropouts occurred (Addorisio et al., 2019; Aranow et al., 2021; Baker et al., 2023; Bellocchi et al., 2023; Bonaz et al., 2016; Clarençon et al., 2014; Genovese et al., 2020; Jensen et al., 2022; Kibleur et al., 2018; Kmet et al., 2020; Koopman et al., 2016; Marrosu et al., 2007; Marsal et al., 2021; Moher et al., 2009; Peterson et al., 2024; Sahn et al., 2023; Sinniger et al., 2020). Table 5 provides a detailed summary of AE, including the total number of AE, the proportion related to VNS, and the occurrence of SAE and dropouts. The percentages were calculated relative to the total number of participants receiving VNS, including those in sham‐controlled groups, as detailed in Table 2.

**TABLE 5:** Summary of VNS adverse events (AE).

Author (year)	Disease	Reported AE	AE severity	Total AE, *N*	Pts with VNS‐related AE, *N*	Total SAE, *N*	Pts with SAE, *N*	Specify SAE	Dropouts, *N*
D'Haens et al. (2023)	CD	teAE: CD exacerbation, abdominal pain, anemia, pyrexia, cachexia, hypokalaemia, pallor, dysphonia, oropharyngeal pain, alopecia, back pain, joint swelling, pain in jaw, and fatigue	Mild/moderate	46	NR	12	1 (6%)	Post‐operative wound infection	1 (6%)
Sinniger et al. (2020)		Voice alteration/hoarseness, and throat pain	Minor	NR	NR	0	0	NA	0
Kibleur et al. (2018)		NR	NA	NR	NR	NR	NA	NA	NR
Bonaz et al. (2016)		Voice alteration/hoarseness, and throat pain	Minor	NR	NR	0	0	NA	0
Clarençon et al. (2014)		NR	NR	NR	NR	NR	NR	NR	NR
Sahn et al. (2023)	IBD	Focal redness and a minor break in the skin	Minor	NR	1 (4.5%)	0	0	NA	0
Peterson et al. (2024)	RA	Implantation‐related AE: implant site hypoaesthesia and inflammation, swelling, incision site swelling, suture related complication, hypoaesthesia, paraesthesia, oropharyngeal pain, rash, scar pain Stimulation‐related AE: Medical device pain and dermatitis contact	Mild	40	11 (18%)	2	2 (3.3%)	Vocal cord paresis and dysphonia	0
Baker et al. (2023)		daAE: Ear pain, medical device discomfort, device inappropriate shock delivery, and scab	Mild to moderate	17	taVNS: 4 (7.5%) Sham: 1 (2%)	0	0	NA	0
Marsal et al. (2021)		Superficial skin abrasion at earpiece site	NR	4	1 (3%)	0	0	NA	0
Drewes et al. (2021)		Transient headache	NR	1	1 (2.8%)	0	0	NA	1 (2.8%)
Genovese et al. (2020)		No daAE/teAE. Surgery‐related events included Horner's syndrome and vocal cord paralysis	NR	NR	0	0	0	NA	0
Addorisio et al. (2019)		No AE reported	NA	0	NA	0	0	NA	0
Koopman et al. (2016)		Fatigue, dysphonia, hypoesthesia, influenza‐like illness, dizziness, nasopharyngitis, nausea, constipation, dyspnea, headache, paresthesia, bradycardia, constipation, dry throat, eructation, nausea, oropharyngeal pain, and postprocedural pain	Mild/moderate	44	9 (50%)	0	0	NA	0
Jensen et al. (2022)	RA or SLE	No AE observed	NA	0	NA	0	0	NA	0
Aranow et al. (2021)	SLE	Observed AE included transient hoarseness and events related to the actual surgical implantation	NR	NR	NR	0	0	NA	0
Bellocchi et al. (2023)	SSc	No AE observed	NA	0	NA	0	0	NA	0
Marrosu et al. (2007)	MS	No serious side effects observed	NR	NR	NR	0	0	NA	0
Kornum et al. (2024)	T1DM (DGP)	Hoarseness, tension headaches, muscular discomfort at the stimulation site, and increased gastrointestinal complaints	NR	NR	1 (1.6%)	0	0	NA	1 (1.6%)
Tarn et al. (2023)	PSS	No serious device‐related AE reported	NR	NR	NR	0	0	NA	1 (2.5%)

Abbreviations: AE, adverse event; CD, Crohn's disease; daAE, device application‐related adverse event; DGP, diabetic gastroenteropathy; IBD, inflammatory bowel disease; MS, multiple sclerosis; N, number; NA, not applicable; NR, not reported; PS, prospective study; PSS, primary Sjögren's syndrome; pts., participants; RA, rheumatoid arthritis; RCT, randomized controlled trials; SAE, serious adverse event; SLE, systemic lupus erythematosus; SSc, systemic sclerosis; T1DM, type 1 diabetes mellitus; teAE, treatment‐emergent adverse event.

## 4 DISCUSSION

### 4.1 Summary of key findings

This systematic review evaluates the therapeutic potential of VNS across a range of autoimmune diseases, including IBD, RA, SLE, SSc, MS, T1DM, and PSS. Findings suggest that VNS holds promise as an adjunctive therapy with the potential to modulate immune responses and alleviate disease symptoms. However, the efficacy and safety profiles vary significantly across studies, reflecting the diverse pathophysiology of these conditions and differences in the mechanisms through which VNS may exert its effects.

The results are consistent with the growing evidence that VNS has anti‐inflammatory and immunomodulatory effects, particularly via the cholinergic anti‐inflammatory pathway (Pavlov & Tracey, 2017; Tracey, 2009). The most robust evidence of efficacy was observed in CD, where several studies demonstrated clinical, biological, and endoscopic remission, suggesting VNS's potential in managing refractory cases of IBD. Similarly, in RA, VNS was associated with significant reductions in pro‐inflammatory cytokines, such as TNF‐α, and improvements in disease activity scores, reinforcing its role as a potential adjunctive therapy.

However, the efficacy of VNS in other autoimmune conditions, such as SLE, SSc, MS, and T1DM, remains less definitive. While some studies reported improvements in symptoms such as pain and fatigue in SLE and SSc, and motor improvements in MS, the overall evidence remains inconclusive, particularly regarding long‐term efficacy and safety. The variability in study designs, VNS protocols, patient populations, and outcome measures highlights the need for more rigorous, standardized research to confirm these preliminary findings.

VNS is thought to exert its effects through the cholinergic anti‐inflammatory pathway, which modulates the inflammatory response by reducing the production of pro‐inflammatory cytokines (Pavlov & Tracey, 2017; Tracey, 2009). While none of the clinical studies in this review explicitly examined the mechanism of VNS in modulating immune responses, this review highlights that VNS, especially when targeting the left cervical vagus nerve, can have significant immunomodulatory effects. The consistent use of left‐sided stimulation in iVNS is a preferable choice to minimize cardiac side effects, given the right vagus nerve's stronger influence on heart rate modulation via the sinoatrial node. This choice reflects a critical safety measure that has become standard practice in VNS applications, based on prior experience with its use in managing epilepsy (Ardesch et al., 2007). Previous studies in epilepsy patients treated with VNS have shown that incubating whole blood with endotoxin results in a significantly reduced release of TNF‐α, IL‐1β, and IL‐6 4 h post‐VNS (Koopman et al., 2016). Similarly, chronic VNS in patients with RA significantly inhibited endotoxin‐induced TNF‐α production in whole blood for up to 84 days (Koopman et al., 2016). These findings suggest that VNS may trigger the release of a soluble factor or prime anti‐inflammatory cells (e.g., acetylcholine‐producing CD4 T cells) in circulation (Rosas‐Ballina et al., 2011).

### 4.2 Safety and adverse events

The safety profile of VNS, as reported in the included studies, is generally favorable, with most adverse events being mild and transient. Commonly reported adverse events, especially in iVNS, included voice alteration, cough, and neck pain. Voice alteration following VNS is attributable to the anatomical pathway and broad functions of the vagus nerve, which innervates the larynx. Since the recurrent laryngeal nerve controls the muscles involved in voice production, VNS can lead to hoarseness, one of the most common adverse effects when VNS is used for the clinical management of epilepsy (Toffa et al., 2020). The noninvasive nature of tVNS was associated with fewer side effects, although data on its long‐term safety remain limited. Surgical risks associated with iVNS, such as infection and device‐related complications, are relatively rare but warrant consideration. The overall tolerability of both iVNS and tVNS in autoimmune populations appears acceptable, but the inconsistent reporting of adverse events across studies limits a comprehensive understanding of their long‐term safety profile.

### 4.3 Notable advantages of the review

Given the emerging evidence for the application of VNS in inflammatory diseases, this systematic review evaluates the therapeutic potential of VNS in autoimmune diseases by critically appraising findings from human clinical studies. There are existing review papers on this topic that provide detailed discussions on the mechanisms of VNS and its potential applications in inflammatory diseases (Cirillo et al., 2022; de Melo et al., 2024; Fang et al., 2023). Cirillo has published an insightful narrative review on the potential mechanisms of VNS in CD and other inflammatory bowel diseases (Cirillo et al., 2022). However, their review is not systematic and does not include or cite the most recent primary clinical studies on VNS in CD, such as those by D'Haens et al. (2023), Sinniger et al. (2020), or Bonaz et al. (2016). Fang et al. have published a comprehensive narrative review on the therapeutic implications of VNS in various disorders (de Melo et al., 2024). However, their review includes both animal and human studies, lacks a systematic approach, does not assess the quality of the studies, and does not follow a framework such as the PRISMA guidelines. Additionally, many of the original clinical studies cited in the present systematic review are not mentioned in their traditional review.

Systematic reviews have the advantage of being rigorous, transparent, and reproducible, leading to more reliable and unbiased conclusions compared to traditional reviews. De Melo et al. recently published a systematic review that includes clinical trials using VNS on serum inflammatory markers such as CRP and cytokines (Fang et al., 2023). While their study is systematic and follows PRISMA guidelines, it does not focus on autoimmune diseases and does not consider the most clinically relevant outcome measures for autoimmune diseases, such as CDAI (for CD) and DAS28‐CRP (for RA). These disease severity scores provide more realistic indications of the usefulness of VNS in clinical practice compared to serum cytokine levels.

We believe that the notable advantage of the present systematic review lies in its focus on human studies involving autoimmune diseases, using relevant clinical outcome measures. It is conducted with a quality assessment and adheres to PRISMA guidelines.

### 4.4 Limitations

This systematic review is subject to several limitations. First, the analysis of stimulation parameters, such as intensity and duration of stimulation periods, was restricted by variations in settings across studies, making it challenging to correlate specific VNS parameters with therapeutic outcomes or adverse events. Second, an exclusive reliance on statistical analysis to assess VNS efficacy may not fully capture the clinical significance of the findings, potentially overlooking meaningful results in case studies that lacked statistical comparisons. Furthermore, the exclusion of non‐English language studies could have introduced bias and reduced the generalisability of the findings. Finally, the heterogeneity in study designs complicates direct comparisons between studies. While categorizing study types and using a standardized quality assessment tool attempted to address this heterogeneity, it remains a challenge to synthesize the overall evidence.

### 4.5 Implications for healthcare practice and future research

VNS shows promise as an adjunctive therapy for autoimmune diseases, particularly in patients who do not respond adequately to conventional treatments. Clinicians may consider the potential benefits of VNS in the future, particularly for specific patient populations with limited therapeutic options. However, before VNS can be routinely integrated into clinical practice, several gaps in the current evidence base need to be addressed. Future research should focus on conducting large‐scale, multicentre RCTs that include diverse patient populations representative of various autoimmune diseases. Standardizing VNS protocols across studies will be crucial for enabling comparisons and establishing best practices for clinical use. Additionally, exploring the mechanistic pathways through which VNS in humans exerts its effects could provide deeper insights into its therapeutic potential. Although much of the mechanistic research has been conducted in animal models, the role of VNS in modulating human immune function remains underexplored. It is not yet known whether the immunomodulatory role of VNS results from stimulation of afferent or efferent vagal fibers. Evidence from experimental animal studies suggests that manipulation of specific neural pathways in the brain can modulate the immune system (Jin et al., 2024). Additionally, there is evidence that systemic inflammation can alter neural firing patterns in the nucleus tractus solitarius (Eftekhari et al., 2020), a finding relevant to the context of autoimmune disease. Whether VNS affects afferent fibers, thereby influencing these regions, or impacts immune cells through efferent fibers (e.g., via splenic innervation) has not yet been investigated in humans and requires further study. In addition, standardizing the reporting of both efficacy outcomes and adverse events in future studies will be essential for a more comprehensive understanding of VNS's safety and effectiveness in autoimmune diseases.

Given the potential costs and adverse effects of VNS, it is crucial to involve multidisciplinary teams in developing clear clinical guidelines, including both indications and contraindications for its application. In this systematic review, we observed that no qualitative or mixed‐methods studies have been published on VNS and autoimmune diseases. Qualitative studies are essential for capturing the lived experiences of patients undergoing VNS, while mixed‐methods studies can integrate clinical evidence from both qualitative and quantitative perspectives. Future investigations could follow the MRC guidelines for complex interventions, incorporating factors such as acceptability, adherence, and fidelity of the intervention (Skivington et al., 2021). This approach would provide a comprehensive understanding of the utility of VNS for patients with autoimmune diseases such as CD and RA, ensuring a holistic evaluation prior to its implementation in healthcare settings.

## 5 CONCLUSIONS

VNS shows promise as an adjunctive therapy for various autoimmune diseases, particularly in patients who do not respond adequately to conventional treatments. The reviewed studies suggest that VNS can modulate immune responses and alleviate symptoms across a range of conditions, including IBD, RA, and SLE. The therapeutic potential of VNS is demonstrated by its effect in reducing pro‐inflammatory cytokines, improving disease activity scores, and enhancing patient‐reported outcomes. However, the efficacy of VNS in other autoimmune diseases such as SSc, MS, and T1DM remains limited and inconclusive. The variability in study designs, patient demographics, and stimulation protocols contributes to mixed results, highlighting the need for more rigorous, standardized research methods. Additionally, while the safety profile of VNS is generally favorable, the inconsistent reporting of adverse events across studies limits a comprehensive understanding of its risk profile, especially for long‐term use. Future research should focus on large‐scale, randomized controlled trials with standardized protocols to better understand the mechanisms, efficacy, and long‐term safety of VNS across different autoimmune conditions. This suggestion has been echoed in previous reports (Cirillo et al., 2022; de Melo et al., 2024; Fang et al., 2023), highlighting the importance of transitioning from proof‐of‐concept studies to large‐scale randomized trials to define the therapeutic potential of VNS for autoimmune diseases and guide its integration into clinical practice. By addressing the current research gaps, particularly in understanding the mechanistic pathways and optimizing stimulation parameters, we can better harness the potential of VNS to improve the quality of life for patients suffering from chronic autoimmune conditions.

## FUNDING INFORMATION

No funding information provided.

## CONFLICT OF INTEREST STATEMENT

None.

## ETHICS STATEMENT

This systematic review does not require ethics approval as it involves the synthesis and analysis of data already available in the public domain, without the collection of primary data or involvement of human participants.

## Supporting information


Appendix S1.

## Data Availability

The data that support the findings of this study are available on request from the corresponding author.
