# Supplementary material for: Assessing the therapeutic potential of vagus nerve stimulation in autoimmune diseases: A systematic review
Source: Physiol Rep. 2025 Feb 4;13(3):e70230. doi: 10.14814/phy2.70230 (PMC11793006; doi:10.14814/phy2.70230)
Supplement: Supplementary file 1 [file PHY2-13-e70230-s001.docx]

Supplementary material S1. Quality assessment scores of included studies using the QualSyst tool.

Study	Q1. Objective	Q2. Design	Q3. Subject selection	Q4. Subject characteristics	Q5. Random allocation	Q6. Blinding investigators	Q7. Blinding subjects	Q8. Outcomes	Q9. Sample size	Q10. Analysis	Q11. Estimate of variance	Q12. Confounding	Q13. Results	Q14. Conclusion	Total Score (%)
Kornum et al. (2024) [34]	2	2	1	2	1	2	2	2	1	2	2	2	2	2	89.3
Peterson et al. (2024) [26]	2	2	1	2	2	2	2	2	NA	NA	NA	2	NA	NA	94.4
Bellocchi et al. (2023) [32]	2	2	1	2	1	1	2	2	2	2	2	2	2	2	89.3
Baker et al. (2023) [27]	2	2	1	2	2	2	2	2	2	2	2	2	2	2	96.4
Sahn et al. (2023) [25]	2	2	1	2	1	1	2	2	2	2	2	2	2	2	89.3
D'Haens et al. (2023) [15]	2	2	1	2	NA	NA	NA	2	1	2	2	1	2	2	86.4
Tarn et al. (2022) [35]	2	2	1	2	1	2	2	2	2	2	2	2	2	2	92.9
Jensen et al. (2022) [30]	2	2	1	2	NA	NA	NA	2	2	2	2	0	2	2	86.4
Aranow et al. (2021) [31]	2	2	1	2	2	2	2	2	2	2	2	2	2	2	89.3
Marsal et al. (2021) [18]	2	2	1	2	NA	NA	NA	2	2	2	2	1	2	2	90.9
Genovese et al. (2020) [28]	2	2	1	2	2	2	2	2	1	2	2	2	2	2	92.9
Drewes et al. (2020) [19]	2	2	1	2	NA	NA	NA	2	2	2	2	1	2	2	90.9
Sinniger et al. (2020) [16]	2	2	1	2	NA	NA	NA	2	1	1	0	1	2	2	72.7
Addorisio et al. (2019) [29]	2	2	1	2	NA	NA	NA	2	2	2	2	1	2	2	90.9
Kibleur et al. (2018) [23]	2	2	1	1	NA	NA	NA	2	2	2	1	1	2	2	81.8
Koopman et al. (2016) [20]	2	2	1	2	NA	NA	NA	2	2	2	2	1	2	2	90.9
Bonaz et al. (2016) [17]	2	2	1	2	NA	NA	NA	2	NA	NA	2	1	2	2	88.9
Clarençon et al. (2014) [24]	2	0	NA	1	NA	NA	NA	2	NA	NA	NA	0	2	1	57.1
Marrosu et al. (2007) [33]	1	0	NA	1	NA	NA	NA	2	1	NA	NA	0	2	2	56.3
